# Association of Taurine with In-Hospital Mortality in Patients after Out-of-Hospital Cardiac Arrest: Results from the Prospective, Observational COMMUNICATE Study

**DOI:** 10.3390/jcm9051405

**Published:** 2020-05-09

**Authors:** Naemi Herzog, Rahel Laager, Emanuel Thommen, Madlaina Widmer, Alessia M. Vincent, Annalena Keller, Christoph Becker, Katharina Beck, Sebastian Perrig, Luca Bernasconi, Peter Neyer, Stephan Marsch, Philipp Schuetz, Raoul Sutter, Kai Tisljar, Sabina Hunziker

**Affiliations:** 1 Department of Medical Communication and Psychosomatic Medicine, University Hospital Basel, 4031 Basel, Switzerland; naemi.herzog@stud.unibas.ch (N.H.); rahel.laager@stud.unibas.ch (R.L.); ebthommen@gmx.net (E.T.); madlainawidmer@gmail.com (M.W.); alessiamichelle.vincent@usb.ch (A.M.V.); annalenakatharina.keller@usb.ch (A.K.); christoph.becker@usb.ch (C.B.); juliakatharina.beck@usb.ch (K.B.); sebastian.perrig@gmail.com (S.P.); 2 Faculty of Medicine, University of Basel, 4031 Basel, Switzerland; stephan.marsch@usb.ch (S.M.); philipp.schuetz@ksa.ch (P.S.); raoul.sutter@usb.ch (R.S.); 3 Emergency Department, University Hospital Basel, 4031 Basel, Switzerland; 4 Institute of Laboratory Medicine, Kantonsspital Aarau, 5000 Aarau, Switzerland; luca.bernasconi@ksa.ch (L.B.); peter.neyer@ksa.ch (P.N.); 5 Department of Intensive Care, University Hospital Basel, 4031 Basel, Switzerland; kai.tisljar@usb.ch; 6 Department of Internal Medicine, Kantonsspital Aarau, 5000 Aarau, Switzerland; 7 Department of Neurology, University Hospital Basel, 4031 Basel, Switzerland

**Keywords:** taurine, metabolomics, out-of-hospital-cardiac arrest, mortality, prognosis

## Abstract

Background: Studies have suggested that taurine may have neuro- and cardio-protective functions, but there is little research looking at taurine levels in patients after out-of-hospital cardiac arrest (OHCA). Our aim was to evaluate the association of taurine with mortality and neurological deficits in a well-defined cohort of OHCA patients. Methods: We prospectively measured serum taurine concentration in OHCA patients upon admission to the intensive care unit (ICU) of the University Hospital Basel (Switzerland). We analyzed the association of taurine levels and in-hospital mortality (primary endpoint). We further evaluated neurological outcomes assessed by the cerebral performance category scale. We calculated logistic regression analyses and report odds ratios (OR) and 95% confidence intervals (CI). We calculated different predefined multivariable regression models including demographic variables, comorbidities, initial vital signs, initial blood markers and resuscitation measures. We assessed discrimination by means of area under the receiver operating curve (ROC). Results: Of 240 included patients, 130 (54.2%) survived until hospital discharge and 110 (45.8%) had a favorable neurological outcome. Taurine levels were significantly associated with higher in-hospital mortality (adjusted OR 4.12 (95%CI 1.22 to 13.91), *p* = 0.02). In addition, a significant association between taurine concentration and a poor neurological outcome was observed (adjusted OR of 3.71 (95%CI 1.13 to 12.25), *p* = 0.03). Area under the curve (AUC) suggested only low discrimination for both endpoints (0.57 and 0.57, respectively). Conclusion: Admission taurine levels are associated with mortality and neurological outcomes in OHCA patients and may help in the risk assessment of this vulnerable population. Further studies are needed to assess whether therapeutic modulation of taurine may improve clinical outcomes after cardiac arrest.

## 1. Introduction

A cardiac arrest is a serious medical condition and often leads to adverse outcomes such as cognitive and functional disabilities and death [1,2,3]. Therefore, early risk stratification is important to decide about further treatment [4]. Different blood markers including metabolomic markers have been proposed to improve risk stratification and decision making [5]. Among these metabolites, the aminoethanesulfonic acid taurine, which is best known as an ingredient in common energy drinks, is an interesting marker for neurological injury. The main quantity of taurine is ingested through animal products such as seafood and meat [6]. However, the human body is also able to synthesize the amino acid through the sulphur-based amino acids methionine and cysteine [7]. It is either excreted through the kidneys or metabolized to taurocholate and passed as bile [7]. Taurine has proven to have favorable effects. Taurine may protect against apoptosis in ischemic myocardiocyte [8], prevent dementia [9] and protect against hypoxia and reoxygenation in neurons [10]. Furthermore, taurine may be directly involved in the pathophysiology of inflammation, oxidative stress, and apoptosis of damaged brain cells [11]. Interestingly, higher plasma taurine concentrations have been shown to improve cardiorespiratory and metabolic functions in patients with sepsis [12,13]. Taurine also has been shown to be increased in patients with a poor outcome after aneurysmal subarachnoid hemorrhage [14,15], unstable angina pectoris or myocardial infarction [16,17]. Lombardini et al. observed that taurine levels in patients with acute myocardial injury were associated with severity of myocardial infarction [18]. Furthermore, elevated blood taurine concentrations were found in patients with ischemic and hemorrhagic stroke [19]. 

Still, there is a lack of studies examining taurine in cardiac arrest patients. Therefore, our aim was to investigate whether taurine levels could be used as a prognostic marker in patients after cardiac arrest. As the destruction of myocardial cells causes an excretion of taurine into the blood stream, we hypothesized that high levels of serum taurine in patients after out-of-hospital cardiac arrest (OHCA) are associated with a higher mortality and poor neurological outcomes.

## 2. Material and Methods

We used prospectively collected patient data from an observational study (COMMUNICATE). The main cohort and a detailed description of the methodology have been published previously [5,20,21,22]. In brief, the study was conducted at the University Hospital Basel, Switzerland and approved by the local Ethics Committee (Northwest and Central Switzerland, No. EKNZ 373/11). All patients included in this observational study or their next of kin gave written informed consent. As an observational study, the research team collected extra blood specimen within the first hours after admission for later measurement of blood markers, collected clinical data of the patients upon admission during hospital stay, and conducted structured interviews. The treatment of patients was up to the treating team. 

Between October 2012 and March 2018, all adult patients admitted with an out-of-hospital cardiac arrest (OHCA) in the intensive care unit (ICU) at the University Hospital Basel were eligible for inclusion. Exclusion criteria included missing measurements of taurine and the absence of consent from either patient or next of kin.

The patients’ medical records were abstracted to collect information regarding patients’ sociodemographics (i.e., age, gender), comorbidities (i.e., diabetes, hypertension, lung-, liver-, kidney- or neurological disease, congestive heart failure, coronary heart disease), risk factors (i.e., smoking status, resuscitation details (i.e., setting of cardiac arrest, initial rhythm, no-flow time (time from cardiac arrest to the beginning of CPR), low-flow time (time from the beginning of CPR to return of spontaneous circulation [ROSC])). In addition, we gathered initial vital signs and blood routine markers upon admission.

The treatment of patients regarding the cardiac arrest was based on the clinical routine in our intensive care unit without interaction with the research team. In 2012, all consecutive patients without complete recovery to premorbid neurofunctional baseline within the first hour following resuscitation were treated with in-hospital systemic cooling via the thermogard XP temperature management system (ZOLL^®^ Medical Corporation, Chelmsford, MA, USA) as a neuroprotectant measure to a target core temperature of 93.2 degrees Fahrenheit (i.e., 34.0 degrees Celsius) for 24 h followed by a rewarming phase with a controlled increase of the core temperature (i.e., 0.2 degrees Fahrenheit or 0.1 degrees Celsius) per hour until 99.5 degrees Fahrenheit (i.e., 37.5 degrees Celsius). Since 2013 (following the TTM-trial (Target Temperature Management) [23]), all consecutive patients without complete recovery were cooled to a target core temperature of 96.8 degrees Fahrenheit (i.e., 36.0 degrees Celsius) for 28 h followed by the rewarming phase using the same thermogard XP temperature management system as mentioned above. Patients with core temperatures below the target temperature were rewarmed with 32.9 degrees Fahrenheit (i.e., 0.5 degrees Celsius) to meet the target core temperatures.

Blood samples of all enrolled patients were taken for routine testing with tube type BD Vacutainer Serum SST II Advance. Additionally, further serum aliquots were frozen at −80 °C and stored until tested for metabolomic markers [24,25,26,27]. As secondary tubes, conical false bottom tubes made of polyethylene with a lamellar plug were used. The AbsoluteIDQ p180 Kit was used for the blood testing, which also includes a MetIDQ™ software (BIOCRATES Life Sciences AG, Innsbruck, Austria). The 96-well-plates were provided by the manufacturer [28,29,30,31,32,33]. Concentrations of taurine were measured using liquid chromatography on an Ultimate 3000 HPLC system (Thermo Fisher, San Jose, CA, USA) coupled to a QTRAP 5500 mass spectrometer (Sciex, Darmstadt, Germany). Preparations of the samples were performed according to the manufacturer’s protocol.

In-hospital mortality was defined as the primary endpoint of this study. The secondary endpoint was a neurological outcome after hospital discharge defined by the cerebral performance category (CPC). No neurological deficit (CPC = 1) and moderate disability (CPC = 2) were classified as favorable neurological outcomes, while severe disability (CPC = 3), coma or vegetative state (CPC = 4) and death (CPC = 5) were determined as poor neurological outcomes [34]. 

Descriptive statistics (i.e., medians and interquartile ranges (IQR) for continuous variables and frequencies for binary or categorical variables) were used to describe the patient cohort. First, we used the Spearman rank test to check for correlations between taurine and different continuous variables. After checking the distribution of the taurine concentration for normality, a log-transformation with base 10 was performed, which converted the skewed distribution into an approximately normal distribution. Second, we used univariable and multivariable logistic regressions analysis to study associations between taurine and the primary and a secondary outcome. We report odds ratios (OR) and 95% confidence intervals (CI). Five models were established for the multivariable analysis, including (1) age and gender, (2) age, gender and comorbidities (i.e., diabetes, hypertension, lung-, liver-, kidney- or neurological disease, congestive heart failure, coronary heart disease, chronic obstructive pulmonary disease, hypertension and malignant tumors), (3) age, gender and initial vital signs, (4) age, gender and blood markers (i.e., troponin, lactate, pH, creatinine) and (5) age, gender and resuscitation measures (i.e., no-flow time, low-flow time, shockable rhythm, bystander CPR). For the univariable models, the receiver operating characteristic (ROC) with the corresponding area under the curve (AUC) was calculated as a measure of discrimination. For graphical display, we divided the taurine levels into quartiles (Q1 < 72.85, Q2 = 72.86–99.4, Q3 = 99.5–153.0, Q4 > 153.1). The quartiles were later used to graph a Kaplan–Meier curve in which the highest quartile was plotted against the lower quartiles. We also performed predefined subgroup analyses regarding gender, comorbidities (coronary heart disease, hypertension, diabetes, smoking) and cause of cardiac arrest (coronary heart disease, respiratory, initial arrhythmia). For the multivariable models, we used multiple imputations for missing covariables as explained in detail in a previous study [35]. A *p*-value < 0.05 was considered statistically significant and all statistical analyses were performed using STATA 15.0.

## 3. Results

### 3.1. Baseline Characteristics

A total of 240 patients with out-of-hospital cardiac arrests and successful resuscitation were included in this analysis. In Table 1, the overall baseline characteristics and stratified by mortality and neurological outcomes (Appendix A) are shown. Patients were predominantly male (72.9%) with a median age of 65 years. Most patients had cardiac comorbidities such as coronary heart disease in 69.0% (*n* = 165), hypertension in 52.3% (*n* = 125) and congestive heart failure in 13.8% (*n* = 33). Furthermore, 50.8% of patients (*n* = 122) were smokers, 26.8% (*n* = 64) had diabetes and 14.2% (*n* = 34) had chronic kidney disease. A total of 130 (54.2%) patients survived until hospital discharge and 110 (45.8%) had a favorable neurological outcome. We found significant differences in age, gender and comorbidities between in-hospital survivors and nonsurvivors. Also, survivors had shorter no-flow and low-flow times, and more often had an initial shockable rhythm and coronary heart disease as cause of cardiac arrest.

### 3.2. Spearman Rank Correlation

In the first step, we investigated correlations between taurine and different clinical parameters. Table 2 shows the results of the Spearman rank test, looking at correlations between taurine and predefined variables including resuscitation measures (no-flow and low-flow time), initial status measures (systolic and diastolic blood pressure, heart rate and respiratory rate) and blood markers (urea, creatinine, lactate, pH and troponin). We found a significant correlation between taurine and low-flow time as well as initial pH. There was no significant correlation between taurine and other lab parameters including troponin. 

### 3.3. Univariable and Multivariable Analysis

Table 3 shows results of univariable and multivariable regression analysis regarding associations between taurine levels with in-hospital mortality and neurologic outcomes.

#### 3.3.1. Association between Taurine and In-Hospital Mortality

The serum concentration of taurine was higher in nonsurvivors than in survivors. After adjusting for age and gender, the taurine concentration was significantly associated with in-hospital mortality (OR 3.93 [95%CI 1.25 to 12.40] *p* = 0.02). Results remained robust after adjusting for comorbidities (OR 4.12 [95%CI 1.22 to 13.91] *p* = 0.02), initial status (OR 5.61 [95%CI 1.62 to 19.41] *p* = 0.01) and resuscitation measures (OR 4.03 [95%CI 1.03 to 15.83] *p* < 0.05). However, there was only low discrimination with an area under the curve (AUC) of 0.57.

#### 3.3.2. Association between Taurine and CPC-Score

The serum taurine levels were higher in patients with poor neurological outcomes compared to patients with a favorable neurological outcome (OR 2.69 [95%CI 0.91 to 7.89] *p* = 0.07, AUC 0.57). Taurine levels were significantly associated with poor neurological outcomes after adjusting for age and gender (OR 3.49 [95%CI 1.11 to 10.92] *p* = 0.03). Results remained robust when additionally adjusting for comorbidities (OR 3.71 [95%CI 1.13 to 12.25] *p* = 0.03) and initial status (OR 4.43 [95%CI 1.28 to 15.25] *p* = 0.02). ROC analysis suggested only low discrimination for the neurological outcome (AUC 0.57).

### 3.4. Association of Taurine in Quartiles and Outcome

**Figure 1:**
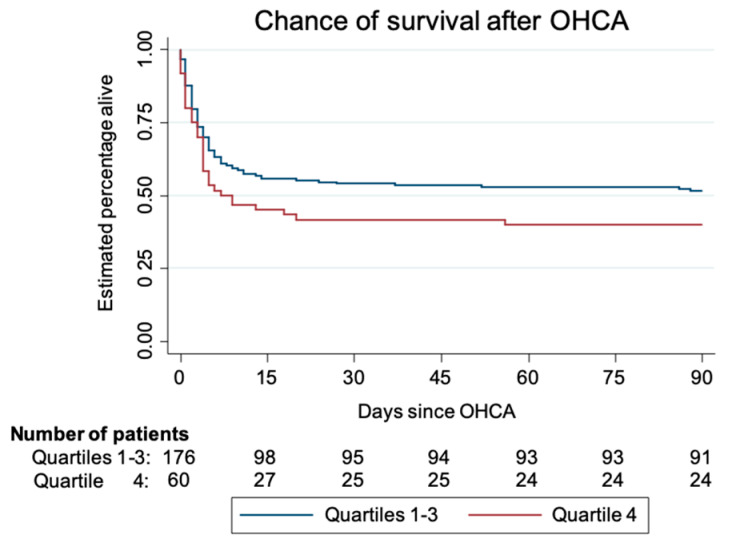
Kaplan–Meier survival.

When stratifying patients according to taurine quartiles (Q1 < 72.85, Q2 = 72.86–99.4, Q3 = 99.5–153.0, Q4 > 153.1), we found a stepwise increase in the OR for mortality, as shown in Table 3, particularly for the third quartile (OR of 1.18) and fourth quartile (OR of 1.83). Figure 1 shows a Kaplan–Meier graph comparing time to death in patients in the highest taurine quartile (>153 pg/mL) compared to the three lower quartiles (*p* log rank = 0.09). 

### 3.5. Subgroups

**Figure 2:**
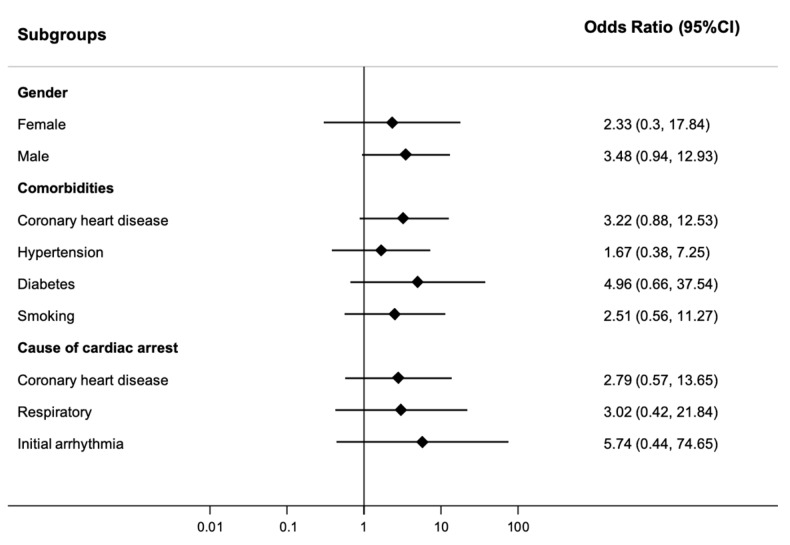
Forest plot for subgroups. Data is presented as odds ratio (OR) and 95% confidence interval (95% CI).

We also performed predefined subgroup analysis to investigate whether the association of taurine and outcome differ among specific patient populations. As shown in Figure 2, there was no evidence for differences in any of the subgroups.

## 4. Discussion

The main findings of this study exploring the prognostic value of taurine levels in OHCA patients is twofold. First, we found that high serum taurine levels were significantly associated with higher in-hospital mortality. The association was particularly relevant in the highest taurine quartile when levels were >153 pg/mL and remained robust in different subgroup analyses. Second, taurine levels were also associated with poor neurological outcomes in this patient population.

Multiple studies have shown a protective effect of taurine in cardiomyocytes and neurons [7,8,10,11]. In our study, higher levels of serum taurine were associated with worse outcomes in regard to survival and neurological outcome, possibly suggesting that the body increases concentrations in response to severe injury. Thus, high taurine concentrations are likely to be a surrogate of an adverse outcome but not causally linked. Furthermore, it is known that the main quantity of taurine is stored in cells and only a small amount is found in body fluids [7]. In addition, in hypoxia or ischemia, brain cells start swelling and release taurine into the blood due to osmoregulatory stress [36,37]. A decrease of taurine in myocardial tissue was observed after damage to rat hearts [38]. Therefore, it is possible that cardiac arrests with longer no-flow and low-flow time led to myocardial and brain tissue destruction and cell swelling, followed by a taurine efflux, and thus an increase in serum concentration. This hypothesis is in accordance with the findings of other studies which showed an increase of taurine concentration after ischemic and hemorrhagic stroke [19]. Similarly, previous cardiological studies demonstrated a correlation between taurine effluxes from cardiomyocytes and severity of cardiac injury [16,17,18,39].

Interestingly, previous research found that high taurine serum concentrations are associated with a good outcome in patients with sepsis [12,13]. The antioxidative action of taurine in septic patients’ blood might counteract the septic progress, and therefore may explain this difference in results. Thus, the prognostic value of taurine levels in OHCA patients that develop septic complications might be limited. As we only measured taurine levels upon admission, we could not assess the prognostic value of taurine over the course of hospitalization in patients developing sepsis in the course of disease. 

The strength of our analysis is the large study population with 255 patients, since most studies looking at taurine only included small patient or animal samples. Our patients are well characterized in regard to clinical and blood work information and we performed a structured outcome assessment. Still, some limitations of this study have been considered. First, we only collected taurine measurements upon admission. Therefore, it is not possible to compare the measures with the patient’s baseline characteristics under noncardiac arrest circumstances or to observe the progression of the taurine concentration as the clinical condition evolves. Second, there is no data about the patient’s diet which could influence taurine levels. Eating a vegan or vegetarian diet might have an influence on the taurine serum levels, as taurine is mostly ingested through animal products. Third, the COMMUNICATE study is a single center study and therefore, the results might not be applicable to the general population. Fourth, some variables could not be measured but had to be estimated and recalled from memory, such as no-flow and low-flow time or bystander CPR. Fifth, the study population is mainly male, however, this might not be relevant since there are no studies showing a difference in the metabolism of taurine between the two genders. Sixth, this is an observational study and thus rather hypothesis generating. Finally, the protocol regarding TTM was changed from hypothermia to the maintenance of normothermia during our study. However, taurine levels were collected upon admission before the start of TTM, and we did not find differences in the prognostic value of biomarkers in regard to TTM.

## 5. Conclusions

Our study found admission taurine levels to be associated with mortality and neurological outcomes in OHCA patients. Taurine may thus help in the risk assessment of this vulnerable population. Further studies are needed to assess whether therapeutic modulation of taurine may improve clinical outcomes after cardiac arrest. 

## Figures and Tables

**Table 1:** Baseline characteristics of the primary endpoint mortality.

		In-Hospital Mortality		
	All	Survivors	Nonsurvivors	*p*-Value
N, %	240	130 (54.2)	110 (45.8)	
Sociodemographics				
Age years, median (IQR)	64.9 (56.6, 74.8)	61.5 (53.1, 73.1)	69.8 (60.5, 77.8)	<0.001
Gender (female), *n* (%)	65 (27.1)	24 (18.5)	41 (37.3)	0.001
Comorbidities and risk factors				
Coronary heart disease, *n* (%)	165 (69.0)	96 (74.4)	69 (62.7)	0.05
Hypertension, *n* (%)	125 (52.3)	66 (51.2)	59 (53.6)	0.70
Congestive heart failure, *n* (%)	33 (13.8)	17 (13.2)	16 (14.5)	0.76
Smoking, *n* (%)	122 (50.8)	77 (59.2)	45 (40.9)	0.01
Diabetes, *n* (%)	64 (26.8)	27 (20.9)	37 (33.6)	0.03
Chronic kidney disease, *n* (%)	34 (14.2)	16 (12.4)	18 (16.4)	0.38
Malignant disease, *n* (%)	23 (9.6)	6 (4.7)	17 (15.5)	0.01
Neurological disease, *n* (%)	24 (10.0)	15 (11.6)	9 (8.2)	0.38
COPD, *n* (%)	19 (7.9)	7 (5.4)	12 (10.9)	0.12
Liver disease, *n* (%)	5 (2.1)	1 (0.8)	4 (3.6)	0.12
Resuscitation measures				
No-flow time, min, median (IQR)	0 (0, 6)	0 (0, 2)	3 (0, 10)	<0.001
Low-flow time, min, median (IQR)	15 (10, 27)	14 (10, 20)	20 (13, 30)	<0.001
Bystander CPR, *n* (%)	153 (64.0)	95 (73.1)	58 (53.2)	<0.001
Shockable initial rhythm, *n* (%)	142 (59.4)	98 (75.4)	44 (40.4)	<0.001
Cause of cardiac arrest				
Coronary heart disease, *n* (%)	120 (50.2)	75 (58.1)	45 (40.9)	0.01
Initial arrhythmia, *n* (%)	46 (19.2)	25 (19.4)	21 (19.1)	0.95
Respiratory, *n* (%)	73 (30.5)	29 (22.5)	44 (40.0)	0.003
Initial status ICU				
Systolic BP, median (IQR)	118 (101, 130)	119 (103, 130)	115 (100, 129)	0.38
Diastolic BP, median (IQR)	67 (55, 78)	70 (58, 79)	63.5 (51, 77)	0.02
Heart rate bpm, median (IQR)	85 (75, 99)	81 (70, 91)	91 (79, 103)	<0.001
Respiratory rate, median (IQR)	17 (14, 20)	17 (14, 19)	16 (14, 20)	0.53
Temperature °C, median (IQR)	35.7 (34.9, 36.3)	35.9 (35.2, 36.4)	35.5 (34.6, 36.2)	0.01
Blood markers				
Initial pH, median (IQR)	7.27 (7.18, 7.33)	7.3 (7.24, 7.34)	7.21 (7.1, 7.3)	<0.001
Initial lactate (mmol/l), median (IQR)	6.3 (3.6, 9.3)	4.6 (3, 6.9)	8.2 (5.6, 10.3)	<0.001
Creatinine (μmol/l), median (IQR)	99 (78, 121)	92.5 (77, 111)	108 (83, 143)	0.00
Troponin(μg/l), median (IQR)	0.33 (0.11, 1.53)	0.31 (0.09, 1.01)	0.36 (0.13, 2.21)	0.12

Data is presented as median (interquartile range) or number (*n*) and percentage (%). IQR: interquartile ranges; CPC: cerebral performance category; COPD: chronic obstructive pulmonary disease; CPR: cardiopulmonary resuscitation; BP: blood pressure in mmHg; bpm: beats per minute.

**Table 2:** Spearman rank correlations of taurine and clinical markers.

	Taurine	
	Rho	*p*-Value
No-flow time	0.04	0.53
Low-flow time	0.17	0.01
BP systolic	−0.09	0.16
BP diastolic	0.02	0.74
Heart rate	−0.02	0.70
Respiratory rate	−0.05	0.46
Urea	−0.03	0.62
Creatinine	0.04	0.57
Lactate	0.08	0.20
pH	−0.13	0.05
Troponin	0.06	0.34

Data is presented as correlation coefficient rho. BP: Blood pressure in mmHg.

**Table 3:** Multivariable regression analysis for mortality and neurological outcome.

	**Survivors, Tau Levels**	**Nonsurvivors, Tau Levels**	**OR (95%CI)**	***p***	**AUC**	**OR (95%CI)** **Age + Gender**	***p***	**OR (95%CI)** **Comorbidities + Age + Gender**	***p***	**OR (95%CI) Initial Status + Age + Gender**	***p***	**OR (95%CI)** **Blood Markers + Age + Gender**	***p***	**OR (95%CI)** **Resuscitation Measures + Age + Gender**	***p***
Tau levels, median (IQR)	94.1 (71.5, 133)	108.5 (74.3, 175)	2.90 (0.99 to 8.53)	0.05	0.57	3.93 (1.25 to 12.40)	0.02	4.12 (1.22 to 13.91)	0.02	5.61 (1.62 to 19.41)	0.01	3.74 (0.96 to 14.49)	0.06	4.03 (1.03 to 15.83)	0.05
Q1, *n* (%)	35 (58.3)	25 (41.7)	1 (reference)			1 (reference)		1 (reference)		1 (reference)		1 (reference)		1 (reference)	
Q2, *n* (%)	37 (60.7)	24 (39.3)	0.91 (0.44 to 1.88)	0.80		0.85 (0.40 to 1.84)	0.68	0.80 (0.36 to 1.79)	0.58	0.98 (0.43 to 2.21)	0.96	1.00 (0.42 to 2.40)	1.00	0.90 (0.36 to 2.25)	0.82
Q3, *n* (%)	34 (55.7)	27 (44.3)	1.18 (0.57 to 2.44)	0.65		1.25 (0.58 to 2.67)	0.57	1.11 (0.49 to 2.51)	0.81	1.57 (0.69 to 3.56)	0.28	1.25 (0.53 to 2.95)	0.61	1.07 (0.43 to 2.69)	0.89
Q4, *n* (%)	26 (43.3)	34 (56.7)	1.83 (0.89 to 3.78)	0.10		2.03 (0.94 to 4.37)	0.07	2.01 (0.89 to 4.53)	0.09	2.53 (1.11 to 5.75)	0.03	1.97 (0.82 to 4.73)	0.13	2.30 (0.89 to 5.94)	0.09
	**Good CPC, Tau Levels**	**Poor CPC, Tau Levels**	**OR (95%CI)**	***p***	**AUC**	**OR (95%CI) Age + Gender**	***p***	**OR (95%CI) Comorbidities + Age + Gender**	***p***	**OR (95%CI) Initial Status + Age + Gender**	***p***	**OR (95%CI) Blood Markers + Age + Gender**	***p***	**OR (95%CI) Resuscitation Measures + Age + Gender**	***p***
Tau levels, median (IQR)	90.3 (71.5, 130)	106.5 (74.3, 171)	2.69 (0.91 to 7.89)	0.07	0.57	3.49 (1.11 to 10.92)	0.03	3.71 (1.13 to 12.25)	0.03	4.43 (1.28 to 15.25)	0.02	3.49 (0.89 to 13.65)	0.07	4.17 (0.97 to 17.90)	0.06
Q1, *n* (%)	30 (50)	30 (50)	1 (reference)			1 (reference)		1 (reference)		1 (reference)		1 (reference)		1 (reference)	
Q2, *n* (%)	32 (52.5)	29 (47.5)	0.91 (0.44 to 1.85)	0.79		0.87 (0.41 to 1.85)	0.72	0.80 (0.36 to 1.76)	0.58	1.02 (0.46 to 2.27)	0.97	1.06 (0.45 to 2.49)	0.89	0.88 (0.34 to 2.25)	0.79
Q3, *n* (%)	29 (47.5)	32 (52.5)	1.19 (0.58 to 2.43)	0.64		1.24 (0.58 to 2.63)	0.58	1.15 (0.51 to 2.59)	0.74	1.48 (0.65 to 3.35)	0.35	1.26 (0.54 to 2.94)	0.59	1.04 (0.40 to 2.68)	0.94
Q4, *n* (%)	21 (35)	39 (65)	1.86 (0.89 to 3.87)	0.10		2.01 (0.93 to 4.35)	0.08	2.00 (0.88 to 4.53)	0.10	2.36 (1.03 to 5.38)	0.04	1.99 (0.83 to 4.80)	0.13	2.64 (0.98 to 7.08)	0.05

Data is presented as number (n) and percentage (%) or median (interquartile range) or odds ratio (OR) and 95% confidence interval (CI). Tau levels: taurine concentration in pg/mL; Q: quartile; p: p-value; AUC: area under the curve.

## Appendix A

**Table A1:** Baseline characteristics of the neurological outcome.

		Cerebral Performance Category		
	All	Good (CPC 1&2)	Poor (CPC 3–5)	*p*-Value
N, %	240	110 (45.8)	130 (54.2)	
Sociodemographics				
Age years, median (IQR)	64.9 (56.6, 74.8)	61.7 (54, 72.9)	69 (58.7, 77.9)	0.004
Gender (Female), *n* (%)	65 (27.1)	17 (15.5)	48 (36.9)	<0.001
Comorbidities & risk factors				
Coronary heart disease, *n* (%)	165 (69.0)	96 (74.4)	69 (62.7)	0.05
Hypertension, *n* (%)	125 (52.3)	66 (51.2)	59 (53.6)	0.70
Congestive heart failure, *n* (%)	33 (13.8)	17 (13.2)	16 (14.5)	0.76
Smoking, *n* (%)	122 (50.8)	77 (59.2)	45 (40.9)	0.01
Diabetes, *n* (%)	64 (26.8)	27 (20.9)	37 (33.6)	0.03
Chronic kidney failure, *n* (%)	34 (14.2)	16 (12.4)	18 (16.4)	0.38
Malignant disease, *n* (%)	23 (9.6)	6 (4.7)	17 (15.5)	0.01
Neurological disease, *n* (%)	24 (10.0)	15 (11.6)	9 (8.2)	0.38
COPD, *n* (%)	19 (7.9)	7 (5.4)	12 (10.9)	0.12
Liver disease, *n* (%)	5 (2.1)	1 (0.8)	4 (3.6)	0.12
Resuscitation measures				
No-flow time, min, median (IQR)	0 (0, 6)	0 (0, 1)	3.5 (0, 10)	<0.001
Low-flow time, min, median (IQR)	15 (10, 27)	12 (8, 20)	20 (13, 30)	<0.001
Bystander CPR, *n* (%)	153 (64.0)	84 (76.4)	69 (53.5)	<0.001
Shockable initial rhythm, *n* (%)	142 (59.4)	88 (80.0)	54 (41.9)	<0.001
Cause of cardiac arrest				
Coronary heart disease, *n* (%)	120 (50.2)	67 (61.5)	53 (40.8)	0.001
Initial arrhythmia, *n* (%)	46 (19.2)	20 (18.3)	26 (20.0)	0.75
Respiratory, *n* (%)	73 (30.5)	22 (20.2)	51 (39.2)	0.001
Initial status ICU				
Systolic BP, median (IQR)	118 (101, 130)	122 (104, 133)	114.5 (100, 128)	0.13
Diastolic BP, median (IQR)	67 (55, 78)	70 (59, 79)	64.5 (54, 77)	0.03
Heart rate bpm, median (IQR)	85 (75, 99)	81 (70, 91)	88.5 (76, 103)	0.003
Respiratory rate, median (IQR)	17 (14, 20)	17 (14, 19)	16 (14, 20)	0.66
Temperature °C, median (IQR)	35.7 (34.9, 36.3)	36 (35.4, 36.4)	35.5 (34.6, 36.2)	<0.001
Blood markers				
Initial pH, median (IQR)	7.27 (7.18, 7.33)	7.29 (7.25, 7.34)	7.23 (7.11, 7.31)	<0.001
Initial lactate, median (IQR)	6.3 (3.6, 9.3)	4.55 (3, 6.5)	7.9 (5.5, 10.1)	<0.001
Creatinine, median (IQR)	99 (78, 121)	95 (77, 113)	102 (81.5, 137)	0.02
Troponin, median (IQR)	0.33 (0.11, 1.53)	0.32 (0.09, 0.99)	0.36 (0.13, 2.06)	0.11

Data is presented as median (interquartile range) or number (*n*) and percentage (%). CPC: cerebral performance category; COPD: chronic obstructive pulmonary disease; CPR: cardiopulmonary resuscitation; BP: blood pressure in mmHg, bpm: beats per minute.
